# Diagnostic value of radiomics and machine learning with dynamic contrast-enhanced magnetic resonance imaging for patients with atypical ductal hyperplasia in predicting malignant upgrade

**DOI:** 10.1007/s10549-020-06074-7

**Published:** 2021-01-20

**Authors:** Roberto Lo Gullo, Kerri Vincenti, Carolina Rossi Saccarelli, Peter Gibbs, Michael J. Fox, Isaac Daimiel, Danny F. Martinez, Maxine S. Jochelson, Elizabeth A. Morris, Jeffrey S. Reiner, Katja Pinker

**Affiliations:** 1 Department of Radiology, Breast Imaging Service, Memorial Sloan Kettering Cancer Center, 300 E 66th Street, New York, NY 10065 USA; 2 Department of Radiology, Memorial Sloan Kettering Cancer Center, 300 E 66th Street, New York, NY 10065 USA; 3 Sloan Kettering Institute, Memorial Sloan Kettering Cancer Center, Mortimer B. Zuckerman Research Center, 417 E 68th Street, New York, NY 10065 USA; 4 Department of Biomedical Imaging and Image-guided Therapy, Division of Molecular and Structural Preclinical Imaging, Medical University of Vienna, Waehringer Guertel 18-20, 1090 Vienna, Austria

**Keywords:** Radiomics, Machine learning, High-risk lesions, ADH, Atypical ductal hyperplasia

## Abstract

**Purpose:**

To investigate whether radiomics features extracted from magnetic resonance imaging (MRI) of patients with biopsy-proven atypical ductal hyperplasia (ADH) coupled with machine learning can differentiate high-risk lesions that will upgrade to malignancy at surgery from those that will not, and to determine if qualitatively and semi-quantitatively assessed imaging features, clinical factors, and image-guided biopsy technical factors are associated with upgrade rate.

**Methods:**

This retrospective study included 127 patients with 139 breast lesions yielding ADH at biopsy who were assessed with multiparametric MRI prior to biopsy. Two radiologists assessed all lesions independently and with a third reader in consensus according to the BI-RADS lexicon. Univariate analysis and multivariate modeling were performed to identify significant radiomic features to be included in a machine learning model to discriminate between lesions that upgraded to malignancy on surgery from those that did not.

**Results:**

Of 139 lesions, 28 were upgraded to malignancy at surgery, while 111 were not upgraded. Diagnostic accuracy was 53.6%, specificity 79.2%, and sensitivity 15.3% for the model developed from pre-contrast features, and 60.7%, 86%, and 22.8% for the model developed from delta radiomics datasets. No significant associations were found between any radiologist-assessed lesion parameters and upgrade status. There was a significant correlation between the number of specimens sampled during biopsy and upgrade status (*p* = 0.003).

**Conclusion:**

Radiomics analysis coupled with machine learning did not predict upgrade status of ADH. The only significant result from this analysis is between the number of specimens sampled during biopsy procedure and upgrade status at surgery.

## Introduction

With the widespread use of image-guided breast biopsies in clinical practice, lesions with uncertain potential of malignancy, also known as high-risk lesions, have become increasingly identified. Several types of high-risk breast lesions exist, with differing upgrade rates at subsequent surgical excisions [1–5]. Atypical ductal hyperplasia (ADH) is a type of high-risk proliferative breast lesion involving the terminal ductal lobular units of the breast and is a non-obligate precursor to invasive breast cancer. At image-guided biopsy, it is difficult to distinguish ADH from low-grade ductal carcinoma in situ (DCIS) [6]. The rate of upgrading ADH to DCIS or invasive cancer has been reported to be between 10 and 31% at a subsequent surgical excision [7].

In clinical practice, the two entities are distinguished pathologically based on quantitative criteria according to the World Health Organization classification of breast tumors [8]. Because the amount of atypia in the biopsy sample may be underestimated at image-guided biopsy, the National Comprehensive Cancer Network guidelines state that not only DCIS requires surgical excision but also ADH [9]. Nevertheless, the majority of ADH end up not being upgraded to malignancy based on surgical excision [10]. Accordingly, a pre-surgical non-invasive tool to identify women at low risk of an upgrade from ADH to DCIS or invasive cancer could serve to obviate surgery for these women, along with the unnecessary associated expenses and morbidity associated with surgery.

A few clinical and technical factors have been reported to predict which patients with ADH at image-guided biopsy are more likely to have an upgrade to malignancy at surgery; these include patient age, lesion size, number of biopsy samples collected, caliber of the needle used for image-guided biopsy, and personal and family history of breast cancer [11–14]. Nevertheless, these factors are not yet enough to change the current recommendation of surgical excision as the current standard of care after ADH is diagnosed at image-guided biopsy [7, 15]. As to imaging features, initial studies involving magnetic resonance imaging (MRI) have shown that no specific imaging feature was able to predict an upgrade for high-risk lesions when detected with MRI [16], and the subsequent upgrade rate for these lesions was between 14 and 38% at surgical excision [17–19].

In this context, artificial intelligence approaches to imaging may present a breakthrough. Several investigators have used various machine learning and computational approaches to predict subsequent upgrades of ADH, using mammographic data, clinical data, and data acquired from biopsy samples [10, 11]. However, to date, no study has applied artificial intelligence to MRI to predict such upgrades. Thus, the aim of the present study was to determine if radiomics analysis coupled with machine learning using MRI data can distinguish which image-guided biopsied lesions with a histological diagnosis of ADH will be upgraded to DCIS or invasive ductal carcinoma (IDC) at surgery. A secondary aim was to determine if qualitatively and semi-quantitatively assessed imaging features, clinical factors, and image-guided biopsy technical factors are associated with upgrade status at surgery.

## Materials and methods

### Study population

This was an institutional review board-approved and Health Insurance Portability and Accountability Act-compliant retrospective study for which the need for written informed consent was waived. This study included patients who underwent state-of-the-art multiparametric MRI with dynamic contrast-enhanced imaging and T2-weighted imaging using a dedicated breast coil, either at our institution or elsewhere, prior to image-guided biopsy (MRI-, ultrasound-, or stereotactic-guided biopsy). We included patients with a suspicious finding on MRI (mass or non-mass enhancement of any size), with or without an ultrasound or mammographic correlate, which on subsequent pathology yielded a diagnosis of ADH (alone or associated with other high-risk lesions) and subsequent surgical excision confirming a benign finding or an upgrade to malignancy. We excluded patients if they underwent a mastectomy for an ipsilateral cancer for which the pathological report was unclear as to which pathological finding was related to the biopsy that yielded ADH.

For all patients, clinical data (patient age, history of breast cancer, presence of ipsilateral or contralateral breast cancer), technical data (caliber of needle used, number of sampled specimens), and pathologic results from the subsequent surgery were collected.

### Breast MRI

Breast MRI examinations were performed on either a 1.5 T or a 3 T scanner using an 8-channel or 16-channel dedicated surface breast coil. Patients underwent state-of-the-art breast multiparametric MRI protocol in agreement with international guidelines [20, 21].

### Imaging analysis

Two fellowship-trained breast radiologists (RLG and KV) with 5 and 2 years of experience interpreted the MR images independently, blinded to patient family and personal history, biopsy results, and pathologic results from the subsequent surgery. Cases in which there was a disagreement between the two readers were re-reviewed by a third reader (CRS) with 6 years of experience to generate a consensus assessment.

On post-contrast-enhanced T1-weighted images, lesion depth (anterior, middle, or posterior depth) was recorded for each lesion as this has been shown to be correlated with malignancy [22]. Morphological features were also assessed according to the Breast Imaging-Reporting and Data System (BI-RADS) lexicon (lesion shape, margin, and internal enhancement characteristics for mass lesions, and distribution and type of enhancement for non-mass enhancements) [23], and readers assigned a BI-RADS classification. Lesion size was measured as the single largest diameter. On T2-weighted and high b-value diffusion-weighted images, signal intensity (hypo-, iso-, hyperintense) and morphology were recorded. Background parenchymal enhancement and fibroglandular tissue were also assessed using maximum intensity projection images and non-fat saturated T1-weighted images, respectively. Time–intensity kinetic curve analysis (signal enhancement in relation to time after contrast injection) was performed using the ROI Enhancement plugin in the OsiriX software [24] by R1. The kinetic curve pattern was described as washout, plateau, or persistent.

### Radiomics analysis

One hundred and one radiomic features were calculated for each patient with CERR software [25], publicly available via GitHub, using MATLAB 2017b (The MathWorks Inc., Natick, MA) and an in-house script was written for batch processing of patient images [14]. The features calculated can be defined as belonging to six classes, based on first-order statistics (22), gray-level co-occurrence matrix (26), run-length matrix (16), size zone matrix (16), neighborhood gray-level dependence matrix (16), and neighborhood gray-tone difference matrix (5), respectively. CERR has recently been shown to conform to the Image Biomarker Standardization Initiative (IBSI) guidelines [26]. Radiomic features were calculated from pre- and post-contrast administration. Delta radiomics, defined as the percentage change in radiomic features between the two timepoints, was also determined. Images were decimated to 32 Gy levels prior to feature calculation.

### Histopathology

Histopathological results from surgical specimens were used as the reference standard. The criteria used to distinguish ADH from DCIS included the presence of at least one of the following two quantitative features according to the World Health Organization classification of breast tumors size limited to 2 mm or smaller and/or involvement of no more than two membrane bound spaces [8].

### Statistical analysis and predictive model building

Univariate analysis using the Chi-square test or Fisher’s exact test was performed to assess associations between imaging features and upgrade status. Differences in lesion size and number of specimens between the two groups were assessed using the Mann–Whitney test. *P*-values < 0.05 were considered significant. To determine inter-reader agreement for qualitatively and semi-quantitatively assessed imaging parameters, Cohen’s Kappa (κ) was estimated. Statistical analysis for the above-mentioned purposes was conducted using SAS version 9.4 software (SAS Institute, Cary, NC, USA).

For radiomic features, data were summarized utilizing medians and inter-interquartile range. Associations between radiomic features and upgrade status were explored using the Mann–Whitney test, with *p*-values < 0.05 regarded as significant. Following univariate analysis, predictive models from radiomic features were created. Correlation analysis was initially employed to remove redundant parameters from advancement to model development, to reduce the possibility of overfitting. If a highly positive (> 0.9) or highly negative (< − 0.9) correlation was noted, the parameter with the lowest area under the receiver operating curve (AUROC) was removed. After parameter selection, undersampling techniques were employed, due to the large imbalance between the majority (no-upgrade) and minority (upgrade) class sizes, to reduce the possibility of any algorithm incorrectly classifying all lesions as belonging to the majority class. Random undersampling at 50% minority class size was utilized for both classes and this process was repeated 1000 times for generalizability. Five-fold cross validation was utilized in place of dedicated train/test datasets and a gaussian support vector machine algorithm was employed. As the current recommendation for lesions diagnosed as ADH at image-guided biopsy is surgical excision, a 50% increased penalty for misclassifying a non-upgraded lesion was utilized. This will have the effect of increasing specificity at the expense of sensitivity to upgraded lesions.

## Results

### Patient population and breast lesion characteristics

**Fig. 1:**
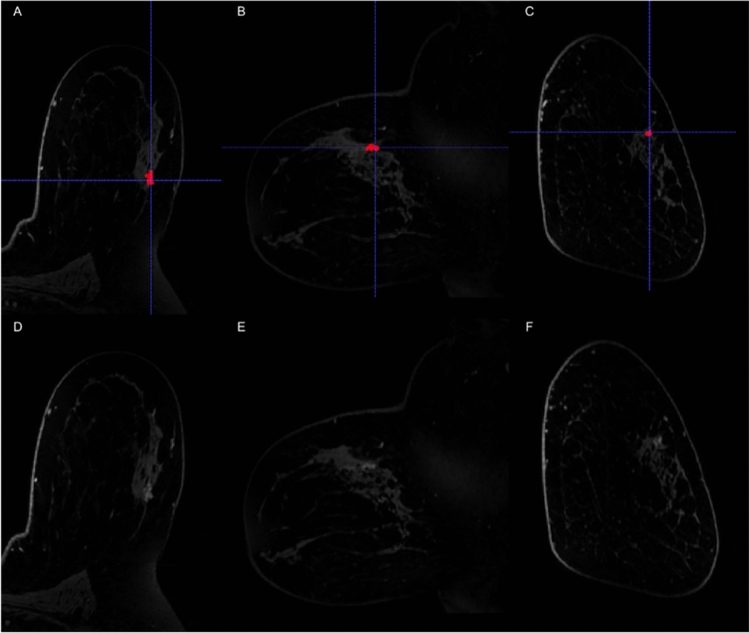
Contrast-enhanced T1-weighted fat-suppressed subtraction maximum intensity projection images in the axial (**a**, **d**) sagittal (**b**, **e**) and coronal (**c**, **f**) planes with (top row) and without segmentation (bottom row), showing a 1.3 cm focal non-mass enhancement in the left upper outer quadrant. MRI-guided biopsy yielded atypical ductal hyperplasia (ADH), ADH diagnosis was confirmed on surgical specimen

This study included 127 patients (average age 51.2 ± 10.2; range 27–78) with 139 lesions, of which 28/139 lesions were upgraded to DCIS or IDC on surgery while 111/139 lesions were not upgraded (Fig. 1). The average lesion size was 15.14 ± 13.23 mm (range 3–70 mm). The majority of lesions (125/139) was assessed with MRI at our institution, while a minority (14/139) was assessed with MRI from an outside institution.

### Radiomics analysis to predict upgrade status

At univariate analysis, 11 radiomic features were found to be significantly different between the two groups (no-upgrade vs upgrade) when utilizing pre-contrast data, 10 radiomic features were significantly different when utilizing percentage change in radiomic features between pre- and post-contrast data, and no radiomic feature was significantly different between the two groups when utilizing post-contrast data. Following correlation analysis, four radiomics features were advanced to model development for both the pre-contrast and delta radiomics datasets. These included 1 first-order feature (Minimum), 1 run-length matrix feature (run-length variance) and 2 neighborhood gray-tone difference matrix features (high dependence high gray-level emphasis and Busyness), findings are summarized in Tables 1 and 2.

**Table 1:** Summary of significant Mann–Whitney U tests for radiomic features created from pre-contrast images

Radiomic feature	No-upgrade	Upgrade	*p*-value
	Median (IQR)	Median (IQR)	
*Skewness (FO)*	− 0.322 (− 0.629 to 0.186)	− 0.580 (− 1.013 to − 0.342)	0.011
Joint average (GLCM)	18.7 (16.2 to 20.3)	19.9 (17.2 to 22.0)	0.025
Sum average (GLCM)	37.3 (32.3 to 40.7)	39.8 (34.5 to 44.0)	0.025
Auto correlation (GLCM)	362 (286 to 434)	412 (313 to 495)	0.029
hglre (RLM)	358 (288 to 422)	397 (313 to 485)	0.036
*lrhgle (RLM)*	462 (377 to 581)	567 (427 to 659)	0.018
*srhgle (RLM)*	333 (266 to 392)	360 (294 to 437)	0.049
hglze (SZM)	347 (284 to 414)	389 (305 to 469)	0.042
*lzhgle (SZM)*	1027 (726 to 1462)	1281 (937 to 1965)	0.040
hgce (NGLDM)	363 (288 to 427)	399 (314 to 490)	0.038
hdhge (NGLDM)	1266 (936 to 1778)	1542 (1209 to 2280)	0.021

Median values with associated interquartile range (IQR) are reported as non-parametric tests were performed. Features selected for advancement to model development are italicized

*FO* first-order, *GLCM* gray-level co-occurrence matrix, *RLM* run-length matrix, *SZM* size zone matrix, *NGLDM* neighborhood gray-level dependence matrix, *hglre* high gray-level run emphasis, *lrhgle* long-run high gray-level emphasis, *srhgle* short-run high gray-level emphasis, *hglze* high gray-level zone emphasis; *lzhgle* large zone high gray-level emphasis, *hgce* high gray-level count emphasis, *hdhge* high dependence high gray-level emphasis

**Table 2:** Summary of significant Mann–Whitney U tests for percentage change in radiomics features between pre- and post-contrast images

Radiomic feature	No-upgrade	Upgrade	*p*-value
	Median (IQR)	Median (IQR)	
*Minimum (FO)*	122 (80 to 228)	184 (111 to 333)	0.041
Sum average (GLCM)	− 16.7 (− 23.9 to − 1.1)	− 21.8 (− 35.4 to − 8.3)	0.047
Auto correlation (GLCM)	− 26.4 (− 38.7 to 0.5)	− 32.3 (− 54.3 to − 12.8)	0.044
lrhgle (RLM)	− 35.7 (− 47.9 to − 13.6)	− 44.8 (− 63.9 to − 22.6)	0.026
*rlv (RLM)*	− 30.0 (− 48.1 to − 5.8)	− 39.2 (− 58.7 to − 24.0)	0.045
lzhgle (SZM)	− 46.7 (− 60.6 to − 20.0)	− 63.8 (− 73.0 to − 39.7)	0.008
hde (NGLDM)	− 21.1 (− 36.8 to − 3.8)	− 30.4 (− 44.4 to − 14.4)	0.047
hgce (NGLDM)	− 31.4 (− 41.6 to − 4.7)	− 38.0 (− 56.8 to − 14.6)	0.047
*hdhge (NGLDM)*	− 42.4 (− 60.6 to − 20.0)	− 61.8 (− 72.9 to − 30.5)	0.006
*Busyness (NGTDM)*	108 (47 to 176)	160 (63 to 206)	0.047

Median values with associated interquartile range (IQR) are reported as non-parametric tests were performed. Features selected for advancement to model development are italicized

*FO* first-order, *GLCM* gray-level co-occurrence matrix, *RLM* run-length matrix, *SZM* size zone matrix, *NGLDM* neighborhood gray-level dependence matrix, *NGTDM* neighborhood gray-tone difference matrix, *lrhgle* long-run high gray-level emphasis, *rlv* run-length variance, *lzhgle* large zone high gray-level emphasis, *hde* high dependence emphasis, *hgce* high gray-level count emphasis, *hdhge* high dependence high gray-level emphasis

Table 3 details the diagnostic performance of the two models. For both the pre-contrast and delta radiomics models, a specificity of around 80% was obtained but at the expense of poor sensitivity (15.3–22.8%). As can be seen, there was a slight improvement in diagnostic accuracy from 53.6% for the pre-contrast radiomics model to 60.7% for the delta radiomics model.

**Table 3:** Summary of predictive models based on pre-contrast images and percentage change in radiomics features between pre- and post-contrast images

	AUROC	Sensitivity (%)	Specificity (%)	PPV (%)	NPV (%)	Accuracy (%)
Pre-contrast	0.514 (0.299–0.728)	15.3 (1.8–42.8)	79.2 (49.2–95.3)	34.8 (11.6–77.3)	48.7 (39.3–56.5)	53.6 (27.5–66.1)
Percentage change	0.540 (0.329–0.752)	22.8 (4.7–50.8)	86.0 (57.2–98.2)	57.5 (22.7–88.4)	53.0 (43.5–60.7)	60.7 (33.9–72.5)

Models were created using gaussian support vector machines (SVMs) and are presented with confidence intervals

*AUROC* area under the receiver operating curve, *NPV* negative predictive value, *PPV* positive predictive value

### Association of qualitatively and semi-quantitatively assessed imaging parameters with upgrade status

Table 4 shows the inter-reader agreement between R1 and R2. For BI-RADS assessment, while there was agreement in 128/139 cases, the κ value of 0.24 showed low agreement, probably due to the low number or BI-RADS category 3 lesions as compared to category 4 lesions. Agreement was moderate for background parenchymal enhancement, T2 and DWI signal intensity, and shape. Agreement was good for fibroglandular tissue, apparent diffusion coefficient signal intensity, and distribution of non-mass enhancement. There was very good agreement for lesion depth within the breast.

**Table 4:** Agreement between reader 1 and reader 2. A *κ* < 0.20 was indicative of poor agreement, *κ* of 0.20–0.40 indicated fair agreement, *κ* of 0.41–0.60 indicated moderate agreement, *κ* of 0.61–0.80 indicated good agreement, and *κ* of 0.81–1.00 indicated very good agreement

Comparison	*κ*	*p*-value
BPE	0.573962	0
FGT	0.736386	0
Depth	0.836347	0
T2	0.427319	3.26E−10
DWI	0.534687	6.79E−11
ADC	0.620233	6.23E−10
BI-RADS	0.247909	9.22E−06
Enhancement type	0.888129	0
Shape	0.569255	1.27E−11
Margins	0.581662	3.44E−10
Enhancement (mass)	0.388235	2.92E−05
Distribution	0.698065	0
Enhancement (non-mass)	0.47231	6.93E−08

*ADC* apparent diffusion coefficient, *BI-RADS* breast imaging-reporting & data system, *BPE* background parenchymal enhancement, *DWI* diffusion-weighted imaging, *FGT* fibroglandular tissue

Table 5 shows the results from univariate analysis according to independent assessments by the two radiologists. Table 6 shows the results from univariate analysis according to consensus assessment. In consensus reading, no significant associations were found between any radiologist-assessed lesion parameter and upgrade status.

**Table 5:** Univariate analysis according to independent radiologist assessment

Imaging feature	Reader 1			*p*-value	Reader 2			*p*-value
	Overall	No-upgrade	Upgrade		Overall	No-upgrade	Upgrade	
BPE				0.11				0.9
Minimal	33 (24)	27 (24)	6 (21)		24 (17)	20 (18)	4 (14)	
Mild	46 (33)	39 (35)	7 (25)		41 (29)	34 (31)	7 (25)	
Moderate	50 (36)	35 (32)	15 (54)		55 (40)	42 (38)	13 (46)	
Marked	10 (7.2)	10 (9)	0 (0)		19 (14)	15 (14)	4 (14)	
FGT				0.6				0.8
Almost entirely fat	2 (1.4)	1 (0.9)	1 (3.6)		1	1 (0.9)	0 (0)	
Scattered FGT	35 (25)	28 (25)	7 (25)		37 (27)	29 (26)	8 (29)	
Heterogeneous FGT	91 (65)	72 (65)	19 (68)		73 (53)	57 (51)	16 (57)	
Extreme FGT	11 (7.9)	10 (9)	1 (3.6)		28 (20)	24 (22)	4 (14)	
Depth				> 0.9				0.9
Anterior	35 (25)	28 (25)	7 (25)		36 (26)	28 (25)	8 (29)	
Middle	74 (53)	59 (53)	15 (54)		70 (50)	57 (51)	13 (46)	
Posterior	30 (22)	24 (22)	6 (21)		33 (24)	26 (23)	7 (25)	
T2 signal intensity				0.9				0.8
Hypointense	2 (1.4)	2 (1.8)	0 (0)		17 (12)	13 (12)	4 (14)	
Isointense	99 (71)	78 (70)	21 (75)		85 (61)	67 (60)	18 (64)	
Hyperintense	38 (27)	31 (28)	7 (25)		37 (27)	31 (28)	6 (21)	
DWI signal (75 lesions)				0.7				0.5
Homogeneous	29 (39)	24 (41)	5 (29)		28 (37)	24 (41)	4 (24)	
Heterogeneous	6 (8)	4 (6.9)	2 (12)		16 (21)	12 (21)	4 (24)	
Rim	1 (1.3)	1 (1.7)	0 (0)		1 (1.3)	1 (1.7)	0 (0)	
No correlate	39 (52)	29 (50)	10 (59)		30 (40)	21 (36)	9 (53)	
ADC signal (61 lesions)				0.4				> 0.9
Hyperintense	4 (6.6)	3 (6.4)	1 (7.1)		4 (6.6)	3 (6.4)	1 (7.1)	
Hypointense	7 (11)	4 (8.5)	3 (21)		10 (16)	8 (17)	2 (14)	
No correlate	50 (82)	40 (85)	10 (71)		47 (77)	36 (77)	11 (79)	
BI-RADS				0.7				> 0.9
3	13 (9.4)	10 (9)	3 (11)		2 (1.4)	2 (1.8)	0 (0)	
4	126 (91)	101 (91)	25 (89)		137 (99)	109 (98)	28 (100)	
Enhancement type				0.6				0.6
Mass like	70 (50)	58 (52)	12 (43)		66 (47)	55 (50)	11 (39)	
Non-mass like	67 (48)	51 (46)	16 (57)		71 (51)	54 (49)	17 (61)	
Mixed	2 (1.4)	2 (1.8)	0 (0)		2 (1.4)	2 (1.8)	0 (0)	
Shape (mass)				0.3				0.2
Oval	19 (26)	18 (30)	1 (8.3)		13 (18)	13 (21)	0 (0)	
Round	16 (22)	12 (20)	4 (33)		19 (26)	15 (24)	4 (33)	
Irregular	38 (52)	31 (51)	7 (58)		42 (57)	34 (55)	8 (67)	
Margins (mass)				0.8				0.5
Circumscribed	30 (41)	25 (41)	5 (42)		36 (49)	32 (52)	4 (3)	
Irregular	34 (47)	29 (48)	5 (42)		32 (43)	25 (40)	7 (58)	
Spiculated	9 (12)	7 (11)	2 (17)		6 (8.1)	5 (8.1)	1 (8.3)	
Enhancement (mass)				0.3				0.4
Homogeneous	28 (38)	25 (40)	3 (25)		37 (49)	33 (52)	4 (33)	
Heterogeneous	35 (47)	29 (47)	6 (50)		38 (51)	30 (48)	8 (67)	
Rim enhancement	8 (11)	5 (8.1)	3 (35)		0 (0)	0 (0)	0 (0)	
Dark internal septations	3 (4.1)	3 (4.8)	0 (0)		0 (0)	0 (0)	0 (0)	
Distribution (non-mass)				0.2				0.3
Focal	23 (33)	20 (37)	3 (19)		26 (35)	22 (39)	4 (24)	
Linear	27 (39)	22 (41)	5 (31)		26 (35)	21 (37)	5 (29)	
Segmental	18 (26)	11 (20)	7 (44)		19 (26)	12 (21)	7 (41)	
Regional	4 (2.9)	1 (1.9)	1 (6.2)		3 (4.1)	2 (3.5)	1 (5.9)	
Multiple regions	0 (0)	0 (0)	0 (0)		0 (0)	0 (0)	0 (0)	
Diffuse	0 (0)	0 (0)	0 (0)		0 (0)	0 (0)	0 (0)	
Enhancement (non-mass)				0.2				0.5
Homogeneous	15 (21)	9 (17)	6 (38)		16 (22)	12 (21)	4 (24)	
Heterogeneous	35 (50)	29 (54)	6 (38)		41 (55)	30 (53)	11 (65)	
Clumped	20 (29)	16 (30)	4 (25)		17 (23)	15 (26)	2 (12)	
Clustered rings	0 (0)	0 (0)	0 (0)		0 (0)	0 (0)	0 (0)	

*ADC* apparent diffusion coefficient, *BI-RADS* breast imaging-reporting & data system, *BPE* background parenchymal enhancement, *DWI* diffusion-weighted imaging, *FGT* fibroglandular tissue

**Table 6:** Univariate analysis according to consensus radiologist assessment

Imaging feature	Upgrade status			*p*-value
	Overall	No-upgrade	Upgrade	
BPE				0.11
Minimal	21 (15)	17 (15)	4 (14)	
Mild	56 (40)	47 (42)	9 (32)	
Moderate	51 (37)	36 (32)	15 (54)	
Marked	11 (7.9)	11 (9.9)	0 (0)	
FGT				0.7
Almost entirely fat	1 (0.7)	1 (0.9)	0 (0)	
Scattered FGT	37 (27)	28 (25)	9 (32)	
Heterogeneous FGT	89 (64)	71 (64)	18 (64)	
Extreme FGT	12 (8.6)	11 (9.9)	1 (3.6)	
Depth				> 0.9
Anterior	36 (26)	28 (25)	8 (29)	
Middle	72 (52)	58 (52)	14 (50)	
Posterior	31 (22)	25 (23)	6 (21)	
T2 signal intensity				0.2
Hypointense	1 (0.7)	0 (0)	1 (3.6)	
Isointense	111 (80)	88 (79)	23 (82)	
Hyperintense	27 (19)	23 (21)	4 (14)	
DWI signal (75 lesions)				0.6
Homogeneous	27 (36)	23 (40)	4 (24)	
Heterogeneous	7 (9.3)	5 (8.6)	2 (12)	
Rim	1 (1.3)	1 (1.7)	0 (0)	
No correlate	40 (53)	29 (50)	11 (65)	
ADC signal (61 lesions)				0.6
Hyperintense	3 (4.9)	3 (6.4)	0 (0)	
Hypointense	9 (15)	6 (13)	3 (21)	
No correlate	49 (80)	38 (81)	11 (79)	
BI-RADS				0.7
3	10 (7.2)	9 (8.1)	1 (3.6)	
4	129 (93)	102 (92)	27 (96)	
Enhancement type				0.6
Mass like	71 (51)	59 (53)	12 (43)	
Non-mass like	66 (47)	50 (45)	16 (57)	
Mixed	2 (1.4)	2 (1.8)	0 (0)	
Shape (mass)				0.12
Oval	16 (22)	16 (26)	0 (0)	
Round	16 (22)	12 (20)	4 (33)	
Irregular	41 (56)	33 (54)	8 (67)	
Margins (mass)				0.6
Circumscribed	34 (47)	30 (49)	4 (33)	
Irregular	32 (44)	25 (41)	7 (58)	
Spiculated	7 (9)	6 (10)	1 (8.3)	
Enhancement (mass)				0.5
Homogeneous	33 (45)	29 (47)	4 (33)	
Heterogeneous	37 (50)	30 (48)	7 (58)	
Rim enhancement	3 (4.1)	2 (3.2)	1 (9)	
Dark internal septation	1 (1.4)	1 (1.6)	0 (0)	
Distribution (non-mass)				0.2
Focal	25 (37)	22 (42)	3 (19)	
Linear	23 (34)	18 (35)	5 (31)	
Segmental	18 (26)	11 (21)	7 (44)	
Regional	2 (2.9)	1 (1.9)	1 (6.2)	
Multiple regions	0 (0)	0 (0)	0 (0)	
Diffuse	0 (0)	0 (0)	0 (0)	
Enhancement (non-mass)				0.9
Homogeneous	10 (15)	7 (13)	3 (19)	
Heterogeneous	36 (53)	28 (54)	8 (50)	
Clumped	22 (32)	17 (33)	5 (31)	
Clustered rings	0 (0)	0 (0)	0 (0)	
DCE (kinetics)*				0.2
Progressive	54 (40)	47 (44)	7 (25)	
Plateau	63 (47)	47 (44)	16 (57)	

*ADC* apparent diffusion coefficient, *BI-RADS* breast imaging-reporting & data system, *BPE* background parenchymal enhancement, *DCE* dynamic contrast-enhanced, *DWI* diffusion-weighted imaging, *FGT* fibroglandular tissue

*Kinetic analysis was performed only by R1

Time–intensity kinetic curve analysis was performed in 135/139 lesions; four lesions were not analyzed due to motion-related artifacts. Progressive contrast enhancement was present in 54 lesions, plateau kinetics was present in 63 lesions, and washout was seen in 18 lesions. There was no association between kinetics and upgrade rate (*p* = 0.2).

### Association between clinical and image-guided biopsy technical parameters

Table 7 shows the results from univariate analysis of clinical and image-guided biopsy technical parameters with upgrade status. The average number of biopsy samples was 8 ± 2.7, and there was a significant correlation between the number of specimens sampled during biopsy and upgrade status (*p* = 0.003). All other parameters were insignificant between the two groups.

**Table 7:** Comparison of clinical and image-guided biopsy technical data between upgraded and no-upgrade patients

Imaging feature	Upgrade status			*p*-value
	Overall	No-upgrade	Upgrade	
Associated malignancy				0.2
Ipsilateral	22 (15.8)	15 (13.5)	7 (25)	
Contralateral	44 (31.7)	34 (30.6)	10 (35.7)	
None	55 (39.5)	48 (43.2)	7 (25)	
History of breast cancer	18 (12.9)	14 (12.6)	4 (14.2)	
Needle caliber				0.9
9 G	123 (88)	99 (89)	24 (86)	
12 G	11 (7.9)	8 (7,2)	3 (11)	
14 G	5 (3.6)	4 (3.6)	1 (3.6)	
Size (mm)	15 (3–70)	9 (3–56)	11 (4–70)	0.077
No. of specimens	8 ± 2.7	9 (2–18)	8 (3–9)	0.003

Size and number of specimens are reported as median values with associated ranges; all other parameters are presented as frequencies, with percentages given in parentheses for each parameter

## Discussion

We undertook this study with the primary hypothesis that radiomics analysis coupled with machine learning using MRI data can distinguish which image-guided biopsied lesions with a histological diagnosis of ADH will be upgraded to a malignant lesion at surgery, but our results showed otherwise. A secondary aim was to determine if conventional qualitatively and semi-quantitatively assessed imaging features, clinical factors, and image-guided biopsy technical factors are associated with upgrade status at surgery. The only significant result from this analysis is between the number of specimens sampled during biopsy procedure and upgrade status at surgery.

In our study, we included MRI scans performed prior to image-guided biopsy showing suspicious enhancement. The upgrade rate at a later surgical excision was 25.5%, which is comparable to that of mammographically detected ADH and unacceptably high to warrant surveillance but also not high enough to justify a costly and invasive surgical procedure for all patients with biopsy-proven ADH. The results published so far on this topic are variable, and to date, no consensus exists regarding the selection of biopsy-proven ADH lesions that may safely undergo observation. A study by Tsuchiya et al. [6] reported that patients with biopsy-proven ADH without suspicious enhancement on breast MRI may be followed up rather than undergo surgical excision, given the high negative predictive value of MRI. This study included only 17 patients (9/17 patients were upgraded to malignancy on surgery) and only looked at post-biopsy MRIs in which it may be difficult to differentiate post-biopsy changes from suspicious persistent enhancement. Another study by Linda et al. [25] included 79 patients with ADH diagnosed on core needle biopsy. The authors reported that cases showing mild or no enhancement on MRI can be followed rather than having surgery. In their study, 8/24 lesions that showed enhancement on MRI were associated with an upgrade on surgical biopsy, whereas only one (1.8%, a low-grade DCIS) of 55 lesions classified as non-suspicious was confirmed to be malignant. Another study by Pediconi et al. [26] assessing 32 high-risk lesions (including ADH) reported that cases of non-suspicious enhancement or no enhancement at breast MRI may undergo follow-up rather than surgery. Although these studies suggest that ADH could be followed with imaging rather than surgically removed in case of no or little contrast enhancement on MRI, another more recent study, also by Linda et al. [27], with a larger sample of 169 high-risk lesions in 166 patients yielded contradicting results: the overall sensitivity, specificity, and positive and negative predictive values of MRI to determine upgrade to malignancy were 72.7%, 74.8%, 30.2%, and 94.8%, respectively. The authors concluded that a negative MRI study warrants follow-up instead of surgery only for lesions with low likelihood of malignancy such as papilloma and radial scar, but it does not help in cases of lobular neoplasia and ADH, and all these latter lesions should be excised.

In our study, MRI-based radiomics analysis coupled with machine learning was not able to accurately predict which biopsy-proven ADH lesions would be upgraded to malignancy at surgery. Although a specificity of around 80% was obtained, this was done at the expense of poor sensitivity. As can be seen, there was a slight improvement in diagnostic accuracy from 53.6 to 60.7% when the radiomics model was based on percentage change in radiomic features between pre- and post-contrast data rather than using only pre-contrast data. Our results involving radiomics analysis are in contrast to a similar study by Ha et al. [10] that included 149 patients who underwent mammography, wherein the convolutional neural network yielded an area under the curve (AUC) of 0.86, sensitivity of 84.6%, specificity of 88.2%, and a diagnostic accuracy of 86.7%. Our results involving radiomics analysis also contradict a study by Cheeney et al. [28] that included 23 high-risk lesions which demonstrated that lesion size and ADC values showed promise for predicting which MRI-detected high-risk lesions will be upgraded to malignancy at surgical excision; in our study, the size of target lesions and radiomic features from diffusion-weighted imaging did not add any value to the machine learning model. Harrington et al. [11] developed machine learning models to predict ADH upgrade in 128 biopsy specimens and concluded that the most important predictors for upgrade status were patient age, size of lesion, number of biopsies, and personal and family history of cancer; however, they did not evaluate imaging features for inclusion into their models. Constant improvements in software and hardware may further improve the accuracy for characterization of high-risk lesions on MRI in the future.

We also found no significant associations between any qualitatively or semi-qualitatively assessed lesion feature on MRI, whether from independent or consensus imaging assessment, and upgrade status (*p*-values ranging from 0.11 to > 0.9). In terms of DCE-MRI features in particular to predict high-risk lesion upgrade risk, our findings agree with the literature which has thus far found morphology and kinetic characteristics to be unpredictive of ADH upgrade to DCIS/IDC at surgery [16, 18, 29, 30].

The lack of significant results involving radiomics or conventional imaging features in our study could be related to the fact that the distinction between ADH and DCIS relies solely on the quantity of atypia present on pathologic specimens (size limited to 2 mm or smaller and involvement of no more than two membrane bound spaces), and thus, it is understandable that imaging features could be similar when comparing two entities that are qualitatively identical. The diagnosis of ADH remains a diagnostic challenge for pathologists, as significant interobserver variability has been reported for both general pathology and breast pathology specialists [31].

Apart from radiomics and conventional imaging-based features, we found that the number of tumor specimens obtained at image-guided biopsy was significantly associated with the upgrade rate. Lesions that were upgraded at surgery had fewer specimens biopsied compared to lesions that were confirmed as ADH at surgery. This is in line with a previous study by Nguyen et al. [32] that showed that incomplete removal of calcifications on stereotactic biopsy (< 95% of the biopsy target) is associated with a higher upgrade rate at surgery. A large retrospective study by Deshaies et al. [12] that included 422 biopsy-confirmed ADH lesions found several independent predictors of an upgrade at surgery, including mammographic lesions, other microcalcifications, and use of a 14G needle. History of ipsilateral or contralateral cancer as well as presence of ipsilateral or contralateral synchronous breast cancer did not affect upgrade rate.

This study has several limitations. The patient cohort used in this study is highly unbalanced (111 patients in the no-upgrade group vs 28 patients in the upgrade group) and while this does not affect univariate analysis using the Chi-square test or the Mann–Whitney test, it may have affected the performance of the predictive models created with machine learning. Future work similar to this may use data balancing techniques such as SMOTE [33] or ADASYN [34] to create synthetic data with the aim of finding more subtle patterns, or more preferably a higher number of patients with the intent to keep datasets balanced. These were, however, not done in this study to keep the methodology clear and rely upon real data from the clinic. The features used as input parameters for the predictive models were chosen based on their performance on the entire dataset, and this can introduce bias and overfitting into the model as features have already been filtered to suit the specific dataset. Even with this possible overfitting, the AUC values for all models fell below 0.700 and would be described as poor [35], suggesting that there are very weak associations to be made at best.

In conclusion, there does not seem to be enough evidence to suggest that we can predict which high-risk lesions will be upgraded to malignancy based on the radiomic data. Our results show, however, that the number of specimens sampled during image-guided biopsy is associated with the upgrade rate of ADH at surgical excision.
